# Deformation-Induced Surface Roughening of an Aluminum–Magnesium Alloy: Experimental Characterization and Crystal Plasticity Modeling

**DOI:** 10.3390/ma16165601

**Published:** 2023-08-12

**Authors:** Yannis P. Korkolis, Paul Knysh, Kanta Sasaki, Tsuyoshi Furushima, Marko Knezevic

**Affiliations:** 1 Department of Integrated Systems Engineering, The Ohio State University, 234 Baker Systems, 1971 Neil Avenue, Columbus, OH 43210, USA; 2 Department of Mechanical Engineering, University of New Hampshire, 33 Academic Way, Durham, NH 03824, USA; 3 Department of Mechanical Engineering, Tokyo Metropolitan University, 1-1 Minami-Osawa, Hachioji-shi, Tokyo 192-0397, Japan; 4 Institute of Industrial Science, University of Tokyo, Komaba 4-6-1, Meguro, Tokyo 153-8505, Japan

**Keywords:** surface roughening, oligocrystal, polycrystal, crystal plasticity, aluminum

## Abstract

The deformation-induced surface roughening of an Al-Mg alloy is analyzed using a combination of experiments and modeling. A mesoscale oligocrystal of AA5052-O, obtained by recrystallization annealing and subsequent thickness reduction by machining, that contains approx. 40 grains is subjected to uniaxial tension. The specimen contains one layer of grains through the thickness. A laser confocal microscope is used to measure the surface topography of the deformed specimen. A finite element model with realistic (non-columnar) shapes of the grains based on a pair of Electron Back-Scatter Diffraction (EBSD) scans of a given specimen is constructed using a custom-developed shape interpolation procedure. A Crystal Plasticity Finite Element (CPFE) framework is then applied to the voxel model of the tension test of the oligocrystal. The unknown material parameters are determined inversely using an efficient, custom-built optimizer. Predictions of the deformed shape of the specimen, surface topography, evolution of the average roughness with straining and texture evolution are compared to experiments. The model reproduces the averaged features of the problem, while missing some local details. As an additional verification of the CPFE model, the statistics of surface roughening are analyzed by simulating uniaxial tension of an AA5052-O polycrystal and comparing it to experiments. The averaged predictions are found to be in good agreement with the experimentally observed trends. Finally, using the same polycrystalline specimen, texture–morphology relations are discovered, using a symbolic Monte Carlo approach. Simple relations between the Schmid factor and roughness can be inferred purely from the experiments. Novelties of this work include: realistic 3D shapes of the grains; efficient and accurate identification of material parameters instead of manual tuning; a fully analytical Jacobian for the crystal plasticity model with quadratic convergence; novel texture–morphology relations for polycrystal.

## 1. Introduction

Deformation-induced surface roughening of a polycrystal is a phenomenon that originates, at least at the mesoscale, from the different crystallographic orientation of the grains. It manifests itself as a gradual change in the surface topography with plastic deformation, resulting in a surface with undulations of increasing amplitude. The resulting irregular, roughened surface can affect both the appearance and performance of a formed part. During forming, the undulations can act as stress risers, triggering an earlier localization of deformation in comparison to the same solid but with a smooth surface, hence limiting the capacity of the solid to stretch in the global sense (e.g., [1]). Furthermore, they can increase the friction between two solids [2] and contribute to, and accelerate, galling and die wear. During service, these surface undulations can impair the fatigue life, acting as nucleation sites for extrusions/intrusions, etc. But even if premature failure is not an issue, the non-smooth surface can cause aesthetic concerns whether in the bare or coated form.

The origins of this phenomenon rest in the strong intrinsic anisotropy of the grains and the presence of preferred crystallographic texture in the as-received material. Therefore, under a macroscopically imposed strain, each grain deforms by activating its own slip systems, which are different from those of differently oriented neighboring grains. At the same time, each grain is forced to accommodate the deformation of its neighbors so that the solid remains a continuum, in the macroscopic sense. However, at a free surface these constraints are much less than in the bulk, leading to more significant differences in individual grain deformation. These differences are macroscopically seen as surface undulations, i.e., deformation-induced surface roughening.

The general aspects of deformation-induced surface roughening have been studied by a number of authors [3,4,5,6,7]. The main result observed in these papers is that the roughness amplitude is linearly proportional to the applied macroscopic, or global, strain. Furthermore, it was also determined that small-scale strain localization at the surface plays a significant role in the overall roughness value. It was also concluded that grain reorientation (the most important source of surface roughening) correlates with the number of active slip systems: highest roughness values were observed in hexagonally close-packed (HCP) metals and lowest in body-centered cubic (BCC) ones. In addition, it was found that the amount of roughness can be significantly affected by microstructural factors, such as the average grain size [8,9,10], as well as grain shape and crystallographic texture [11,12,13].

Research on surface roughening has been performed for many metals, including pure aluminum and aluminum alloys [14,15,16,17,18,19,20,21,22], titanium [23], steel [22,24,25], copper [26,27,28], nickel [29], tantalum [30], magnesium [31,32] and others. The most common testing technique is the uniaxial tension test [5,16,22]. Evolution of roughness during equibiaxial and plane-strain tension using a Marciniak test has been studied, as well [24,33]. Roughening during forming, for example, cup drawing [26,27], bending [32] or drawing through dies [21], has also been investigated.

In order to model the physics of deformation at the mesoscale and above, crystal plasticity models are most commonly used [3,18,19,20,23,34,35]. Most of these studies involve a synthetic, i.e., artificial, grain morphology, generated using procedures such as Voronoi tessellation [36,37]. Only a few surface roughening studies have attempted to reconstruct the exact arrangement of gains in a given specimen. In [20,29,30,38], oligocrystal specimens that had one layer of grains through the thickness were considered; in these works, the grain shapes were, or were assumed to be, columnar, i.e., the 3D grains were obtained by extruding through the thickness the 2D grain shapes seen in one of the outer faces. 

In the present study, we also work with an oligocrystal containing one layer of grains. We observed that in our specimens most of the grains were strongly non-columnar. To tackle this, a reconstruction procedure that is based on a morphing approach was developed earlier [39]. This made it possible to incorporate into this work realistic (i.e., non-columnar) shapes of the grains, based on 2D scans obtained from the top and bottom faces, in contrast to the previous efforts described above. Since the mesoscale oligocrystal specimen described in the following sections contains only a few grains, we suggest that having realistic geometries of the grains (in contrast to artificial/simplified geometries) is beneficial for capturing the physics of the deformation accurately. In addition, modeling the behavior of a realistic texture as accurately as possible and comparing it to experiments is one of the main objectives of the current study, as it allows us to assess the predictive capabilities of the Crystal Plasticity Finite Element (CPFE) model we used.

In the following sections, we describe the details of the oligocrystal specimen preparation, experimental setup, formulation and calibration of the CPFE model and the Finite Element (FE) mesh generation. Subsequently, we analyze and compare with experimental data the following features: the deformed shape of the oligocrystal specimen; the surface topographies at the top and bottom faces; derivatives of the elevation; the evolution of average roughness value with straining; the texture and Schmid factor after deformation; and the reorientation of several soft and hard grains of the specimen. In addition, we further validate our CPFE model using data from a polycrystal experiment. Specifically, we construct two artificial Representative Volume Elements (RVEs) of the same crystallographic texture and grain size distribution as the actual specimen and simulate the evolution of the average roughness value. Subsequently, we use the same experimental data for discovering relations between the local texture and the elevation of a given grain. These relations provide an alternative interpretation of the surface roughening phenomenon based on the difference in hardness of neighboring grains and, more specifically, on the difference in their Schmid factors. We point out that our study is at the mesoscale. One could argue that roughening originates at the atomic [40] or dislocation length scales [41]. For example, at the dislocation length-scale, the annihilations of dislocations on the sample surface leave surface steps whose magnitude is proportional to a Burgers vector magnitude, thus making an initially smooth free surface rough. In this way, even a single crystal can roughen. This is beyond the scope of this work; as will be shown, while our experiments reveal significant surface roughening, the resolution of our surface measurements is not sufficient to also capture dislocation-scale contributions.

In comparison to the state of the art, the present work considers realistic 3D shapes of the grains: since we model the behavior of an actual, non-columnar oligocrystal, it is crucial to work with 3D grain morphologies, instead of extruding a surface scan through the thickness. This accurate volumetric reconstruction is accomplished here by a procedure described and verified in [39]. Also, the adopted crystal plasticity model uses a fully analytical Jacobian, guaranteeing quadratic convergence, and integrates a dislocation density-based hardening law, which was verified to work well for a number of metals (e.g., [42,43,44]). The model is evaluated here for the first time for the prediction of roughness development with plastic strain. The identification of the material parameters is carried out efficiently and accurately [45], with the cost function explicitly defined and minimized, instead of a tedious manual parameter tuning. Finally, novel texture–morphology relations are obtained here for the polycrystal case.

## 2. Experiments

### 2.1. Opening Remarks

The goals of this work are to study the roughening behavior of an aluminum alloy and to assess the performance of an advanced computational model by comparing its predictions to experiments. It is desired to study the simplest possible problem, and to have a model of as high a fidelity as possible, to be able to highlight its successes and pinpoint its limitations. Therefore, it was decided to study the (macroscopically) uniaxial tension on a flat specimen. This geometry enables easy observations of surface roughening. In selecting the most suitable specimen, a polycrystal could be considered. Considering that such a specimen is used not only for texture measurements but also for subsequent testing and surface topography analysis, some type of non-destructive, 3D scanning would need to be performed to obtain its exact grain arrangement and texture, which is a challenging task. In addition, the resulting model would contain a significant number of finite elements, making it computationally very expensive. Instead, an oligocrystal specimen that contains relatively few grains arranged in a single layer was selected, avoiding both of these problems.

### 2.2. Specimen Preparation

**Figure 1:**
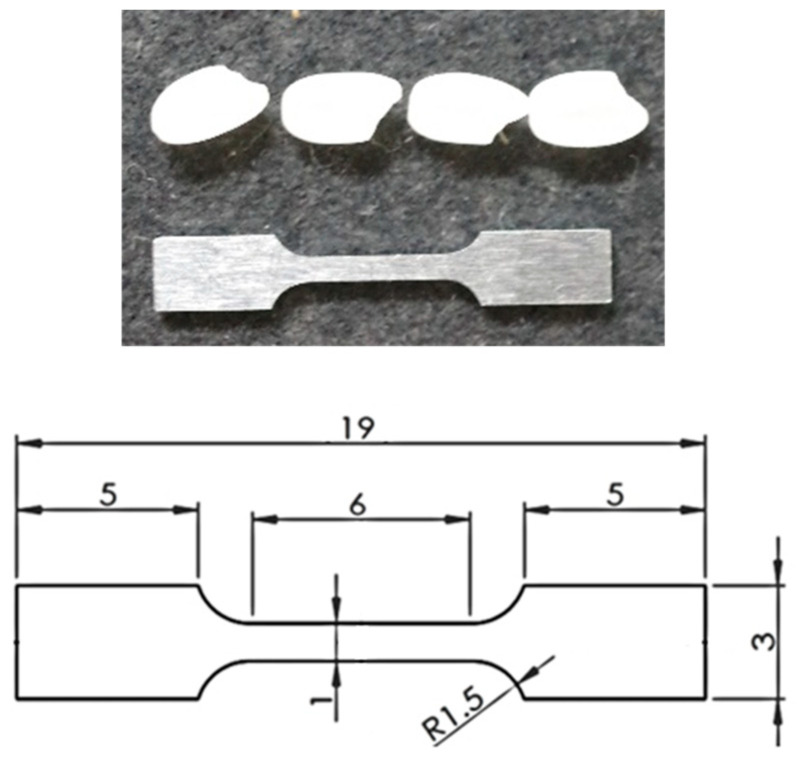
Geometry of the oligocrystal specimen. (**Top**) Photo showing the relative size of the dogbone specimen in comparison to four rice grains on the top. (**Bottom**) Engineering drawing of the dogbone specimen (dimensions in mm). The thickness is 134 μm.

The material of this study is the commercially available aluminum alloy AA5052-O, which contains 95.7–97.7 wt. % aluminum and 2.2–2.8 wt. % magnesium [46]. The as-received sheet shows a typical rolling texture, see Appendix A. The average grain diameter of the polycrystalline AA5052-O was calculated using the ASTM standard E112-13 [47] and found to be 10.3 μm. In order to obtain the desired mesoscale oligocrystal specimen, the following procedure was carried out:

1. An AA5052-O sheet of 0.5 mm thickness is prestrained in uniaxial tension to 10% nominal strain.
2. The plate is heat-treated at 450 °C for 1 h.
3. Tensile specimens of the geometry shown in Figure 1 are cut from the plate.
4. The tensile specimen thickness is reduced from 0.5 mm to 0.134 mm by polishing.
5. The top and bottom faces of the tensile specimen are polished, in preparation for the tensile experiment.

The 4th step was deemed necessary as it was not possible to find a combination of prestraining and heat-treating such that only one layer of grains would appear in the final specimen of 0.5 mm thickness. Therefore, a cycle of scans (see below) and polishing were performed in sequence, until the same grains appeared on both faces. Of course, this process is limited by the increasing fragility of the resulting specimen with every grinding iteration, and so it was interrupted when an acceptable compromise between identical grains and specimen fragility was found. A challenge in these experiments is that if a “soft” grain happens to be in the neighborhood of the specimen shoulders, then during tensile testing only that grain will deform, leaving the rest of the specimen almost intact. Hence, multiple iterations of the process described here were performed, until a number of specimens that provided useful results were identified.

### 2.3. Experimental Setup

**Figure 2:**
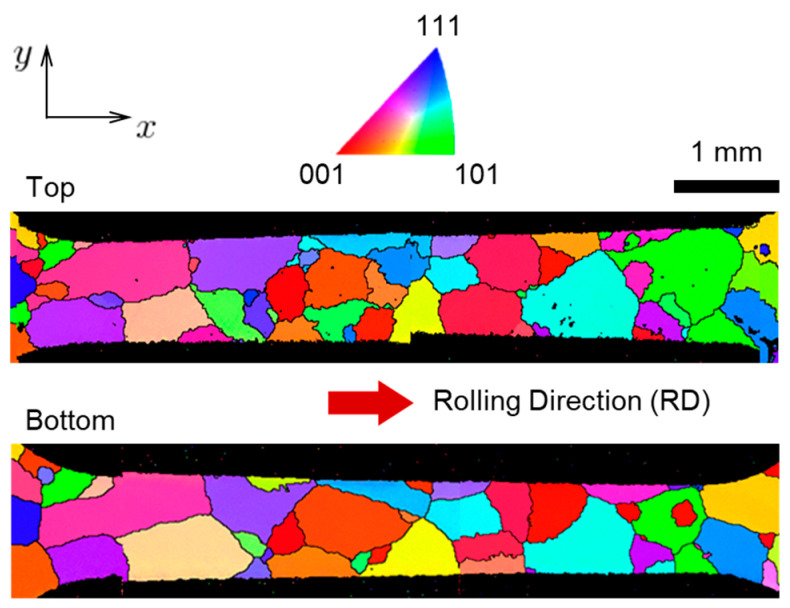
Initial grain structure of the oligocrystal specimen with Inverse Pole Figure (IPF) colors representing grain orientations relative to the sample Rolling Direction (RD) according to the colors in the IPF triangle. The specimen is cut along the RD. Scans of top and bottom faces are shown. The scan of the bottom face was flipped horizontally for ease of interpretation.

The resultant oligocrystal specimen contains relatively few grains: 38 are those that can be seen on both top and bottom faces of the specimen. For the purposes of this work, it is very important that these grains form one thin layer. This allows us to reconstruct the volumetric grain morphology based only on the planar morphologies visible at the top and bottom faces, i.e., no 3D scanning is needed. A specialized procedure for performing such a reconstruction was developed earlier and its accuracy was verified [39]. To allow scanning of the texture, the specimen was prepared using the following sequence—#1200 Emery polishing, 3 μm diamond grinding, acetone washing, electrolytic polishing (60% HClO4:ethanol = 4:45 volume ratio). The actual texture scanning at the top and bottom faces was performed using a HITACHI S-3700N Scanning Electron Microscope (SEM, Tokyo, Japan) with a Nordlys NL 04-2201-03 Electron Back-Scatter Diffraction (EBSD, Abingdon, UK) system. The observation magnification of the SEM is 30×, and the step size is 10 μm. A clean-up process was performed once to reduce the noise. The shapes and orientations of grains obtained from these scans are shown in Figure 2, and the corresponding Euler angles are provided in Table 1. It can be seen that most of the grains form one layer through the thickness, except for relatively few grains that appear either only at the top or only at the bottom and do not go through.

The oligocrystal specimen was then plastically deformed in uniaxial tension. A Zwick/Roell Universal Testing Machine (Ulm, Germany) of 5 kN force capacity and equipped with a laser extensometer laserXtens was used. The specimens were tested under displacement control, at a nominal strain rate of 5 × 10^−4^ s^−1^. The resultant nominal strain in the gauge region of the specimen is ~10%. This value of strain induces a vivid, observable change in the surface topography. At the same time, this strain is small enough to avoid necking or fracture.

The surface topography was obtained with a KEYENCE VK-X100 laser confocal microscope (Osaka, Japan). The lateral resolution of the microscope is 0.01 μm and the vertical resolution is 0.005 μm [48]. This allows scanning of the entire gauge region of the deformed specimen. The data obtained are an array of points that can be visualized as a 3D or contour plot for further comparison and analysis.

## 3. Modeling

### 3.1. Crystal Plasticity Finite Element (CPFE) Model Overview

In order to capture the physics of crystal plasticity, we used the crystal plasticity-based constitutive law originally developed in [49], and later advanced with dislocation–density-based hardening [50]. This model represents purely mechanical effects, e.g., not deformation-induced heating [51] or other effects, which is an acceptable assumption for this aluminum alloy. For completeness, in this section we summarize this model.

The total deformation gradient F can be decomposed into elastic (F*) and plastic ($F^{p}$) components:

(1) $$F=F\text{*}F^{p}$$

The elastic component F* is related to stress by Hooke’s law:

(2) $$T\text{*}=CE\text{*}\;\mathrm{or}\;T\text{*}={F\text{*}}^{-1}\;\left[\;\left(\det F\text{*}\right)\sigma \;\right]\;F^{\text{*}-T}\;\mathrm{with}\;E\text{*}=\frac{1}{2}\left({F\text{*}}^{T}F\text{*}-I\right)$$

where T* and E* are the Piola–Kirchhoff stress tensor and Green–Lagrange finite strain tensor, respectively (that form work-conjugate stress and strain measures), C is the 4th-order elasticity tensor and ***σ*** is the Cauchy stress tensor.

The evolution of plastic components of the deformation gradient due to slip is given by:

(3) $${\dot{F}}^{p}=L^{p}F^{p}$$

where $L^{p}$ is the plastic velocity gradient, that can be represented as:

(4) $$L^{p}=\underset{\alpha }{\sum }\;{\dot{\gamma }}^{\alpha }\;b_{0}^{\alpha }⊗n_{0}^{\alpha }$$

In this equation, the vectors $b_{0}^{\alpha }$ and $n_{0}^{\alpha }$ indicate the slip-direction and the slip-plane normal, for a given slip system *α*. The term ${\dot{\gamma }}^{\alpha }$ is the shearing rate of slip system *α*, that can be expressed with the following power-law relationship:

(5) $${\dot{\gamma }}^{\alpha }={\dot{\gamma }}_{0}\;{\left(\frac{\;\left|\tau^{\alpha }\right|\;}{\tau_{c}^{\alpha }}\right)}^{1/m}sign\left(\tau^{\alpha }\right)$$

where ${\dot{\gamma }}_{0}\;$= 0.001 s^−1^ is a reference slip rate and m = 0.05 is the power-law exponent. A detailed study of this exponent was conducted in [52]. Based on those findings, and in the absence of an experimental study of strain-rate sensitivity for the material at hand (e.g., [53,54]), the value of 0.05 was selected, ensuring proper selection of active slip systems for accommodating the imposed plastic strain but not affecting strain-rate sensitivity. 

In Equation (5), $\tau^{\alpha }$ is the resolved shear stress and $\tau_{c}^{\alpha }$ is the characteristic resistance shear stress. The latter is often assumed to be composed of 4 different contributions:

(6) $$\tau_{c}^{\alpha }=\tau_{o,f}+\tau_{o,HP}+\tau_{for}^{\alpha }+\tau_{sub}=\tau_{o}+\tau_{for}^{\alpha }+\tau_{sub}$$

The four terms in Equation (6) represent different physical aspects: $\tau_{o,f}$ is a friction term also called Peierls initial slip resistance, $\tau_{o,HP}$ is a barrier-effect term and $\tau_{for}^{\alpha }$ and $\tau_{sub}$ are the contributions from interaction of dislocations. While the first two terms are fixed, the latter two evolve with the forest and substructure dislocation density evolution. Notice that from the four mechanisms that affect $\tau_{c}^{\alpha }$, only one is slip system dependent. The term $\tau_{o,HP}$ is expressed using the Hall–Petch relationship [55,56]:

(7) $$\tau_{o,HP}=\mu \;H\sqrt{\frac{b}{d_{g}}}$$

where μ is the shear modulus (26.1 Gpa), H = 0.136 is the Hall–Petch parameter, b is the Burgers vector (0.286 nm) and $d_{g}$ = 10.3 μm is the average grain diameter of the polycrystalline AA5052-O, see Section 2.2. With these inputs, Equation (7) evaluates to 18.7 Mpa for this material. However, since the material is not experimentally characterized in terms of a variable grain size, the Hall–Petch term is omitted in this work. Instead, the Peierls initial slip resistance is increased accordingly, to represent the total non-evolving initial value of slip resistance as $\tau_{o}=\tau_{o,f}+\tau_{o,HP}$, see the 2nd equality in Equation (6). The terms $\tau_{for}^{\alpha }$ and $\tau_{sub}$ are obtained from the following relations:

(8) $$\tau_{for}^{\alpha }=\chi \;\mu \;b\;\sqrt{\rho_{for}^{\alpha }}$$

(9) $$\tau_{sub}=k_{sub}\;\mu \;\sqrt{\rho_{sub}}\;\log\left(\frac{1}{b\;\sqrt{\rho_{sub}}}\right)$$

where $\rho_{for}^{\alpha }$ and $\rho_{sub}$ are the corresponding dislocation densities, χ = 0.9 is a dislocation interaction parameter and $k_{sub}$ = 0.086 is a parameter that ensures that Equation (5) recovers the Taylor law at low dislocation densities.

The last two equations contain non-proportional relationships between two of the components of the characteristic resistance shear stress and the forest and substructure dislocation densities. The forest density $\rho_{for}^{\alpha }$ evolves in the following way:

(10) $$\frac{\partial \rho_{for}^{\alpha }}{\partial \gamma^{\alpha }}=k_{1}\;\sqrt{\rho_{for}^{\alpha }}-k_{2}\;\rho_{for}^{\alpha }$$

where $k_{1}$ is a coefficient for the rate of dislocation storage (also called trapping-rate coefficient, see Table 2) and $k_{2}$ is the coefficient for the rate of dynamic recovery. In turn, these two coefficients follow the relation:

(11) $$\frac{k_{2}}{k_{1}}=\frac{\chi \;b}{g}\;\left[1-\frac{k\;T}{D\;b^{3}}\;\ln\left(\frac{\dot{\varepsilon }}{{\dot{\varepsilon }}_{0}}\right)\right]$$

where k = 1.38 × 10^−23^ J K^−1^, ${\dot{\varepsilon }}_{0}$ = 0.001 s^−1^, g, D and T = 298 K are the Boltzmann’s constant, the reference strain rate, an activation barrier, the drag stress and the room temperature, respectively. Also, $\dot{\varepsilon }\;$ is the macroscopic (i.e., global) strain rate. The substructure density increase is proportional to the rate of dynamic recovery of all active dislocations via:

(12) $$\Delta \rho_{sub}=q\;b\;k_{2}\;\sqrt{\rho_{sub}}\underset{\alpha }{\sum }\;\rho_{for}^{\alpha }\;\left|\Delta \gamma^{\alpha }\right|$$

where q is a fitting coefficient representing the fraction of *α*-type dislocations that do not annihilate but form substructures.

The initial value of forest density $\rho_{for}^{\alpha }$ is set to 10^10^ m^−2^, typical for annealed FCC metals. The substructure density $\rho_{sub}$ theoretically initiates from 0, but numerically starts from the value of 0.1 m^−2^. Both densities subsequently evolve according to Equations (10) and (12). It should be stated clearly that we do not have measurements of dislocation density to compare these predictions to. However, they are comparable to predictions in other works [57,58].

**Table 2:** Material parameters of AA5052-O alloy.

Parameter	Symbol	Value	Unit	Comment
Aluminum crystal elastic constants	$C_{1111}$	107,300	Mpa	[59]
	$C_{1122}$	60,900	Mpa	[59]
	$2C_{2323}$	56,600	Mpa	[59]
Initial slip resistance	$\tau_{0,f}$	51.3	Mpa	Optimization
Shear modulus	μ	26,100	Mpa	[58]
Burgers vector	b	0.286	nm	[58]
Trapping rate coefficient	$k_{1}$	6.376 × 10^7^	m^−1^	Optimization
Activation barrier	g	0.005283	-	Optimization
Drag stress	D	1535	Mpa	Optimization
Dislocation recovery rate	q	16	-	[58]

### 3.2. Identification of Material Properties

The crystal plasticity framework described above is implemented in a user-material subroutine (UMAT) that is called within Abaqus/Standard (implicit). The UMAT needs access to texture information (Euler angles for each individual grain), as well as material properties. Many of these properties, such as the elastic constants $C_{ijkl}$, shear modulus μ, Burgers vector b and dislocation recovery rate q are reported in the literature, for the present or similar alloys (Table 2). However, four material constants that enter Equation (6) need to be identified for our specific material (AA5052-O).

**Figure 3:**
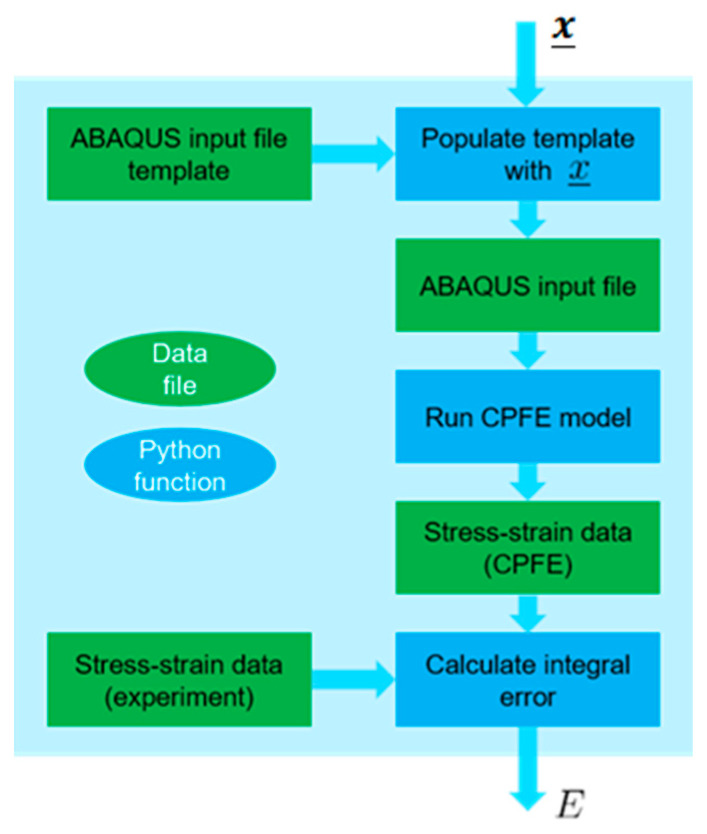
A schematic of the procedure of formulating the objective function used in optimization procedure, where $\underline{x}$ is the vector of input parameters and *E* is the scalar error value (output).

Apart from specialized methods [60], the identification of unknown material parameters is often carried out manually by trial and error—until the response of the model (for a chosen loading case) matches the corresponding experimental response. Since in our case every trial is associated with running a CPFE simulation, this could be an acceptable method for identifying one unknown parameter, however, it becomes very tedious and inefficient when we deal with four parameters. Therefore, we automated the material identification problem by considering it as an optimization problem. The corresponding objective function is a Python function assembled from several stages, as illustrated in Figure 3. This function reads a vector $\underline{x}$ of the four unknown parameters as an input and runs an FE simulation of uniaxial tension of a polycrystal block made out of 16^3^ = 4096 C3D8 elements (continuum, linear, full integration, with selectively reduced integration on the volumetric terms to avoid volumetric locking), where each element corresponds to a single crystal. The size of this domain is statistically significant, so that the macroscopic predictions are not influenced appreciably [61]. The experimentally measured crystallographic texture of the polycrystal (see Appendix A) is used in the FE simulation. Subsequently, the objective function compares how well the corresponding nominal stress–strain curve matches with the experimental response of the corresponding polycrystal and returns the integral error ($E=\int_{0}^{\epsilon }\left|\sigma_{FE}-\sigma_{exp}\right|d\epsilon$) between these two curves. The optimization goal is to minimize the value of E. 

**Figure 4:**
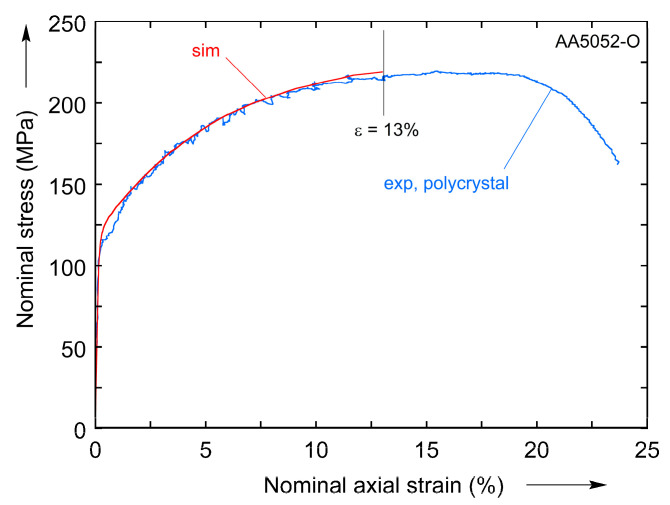
Measured and predicted nominal stress–strain curves for a polycrystal, that are used for calculating corresponding error value ($E=\int_{0}^{\varepsilon }\left|\sigma_{FEA}-\sigma_{exp}\right|d\epsilon$). The plot shows the match between two curves after the 4 unknown parameters were identified.

In order to solve this optimization task, we used “blackbox” [45]—an optimization method specifically designed for black-box functions (ones that have input–output nature, instead of being explicitly formulated) that are expensive to evaluate. The method was successfully used previously for identification of post-necking hardening behavior of stainless steel across a range of strain rates and temperatures in a fully coupled way [62]. Since the method itself is also implemented as a Python module, it can be easily integrated into the current problem. The values of the four material constants identified in this way are listed in Table 2. The final matching obtained between the experiment (on as-received, polycrystalline AA5052-O) and the FE model prediction with the optimized material parameters is shown in Figure 4. The total number of objective function evaluations (as described in Figure 3) used is 150, i.e., after this number is reached, the current solution (i.e., the current set of the four material constants) is taken as the optimum. This solution is verified by running the optimization procedure a few times. Since the procedure involves random numbers, each new run starts with different initial estimates. The same result is obtained consistently, indicating that the optimization algorithm is not trapped in local minima.

### 3.3. Mesh Construction

**Figure 5:**
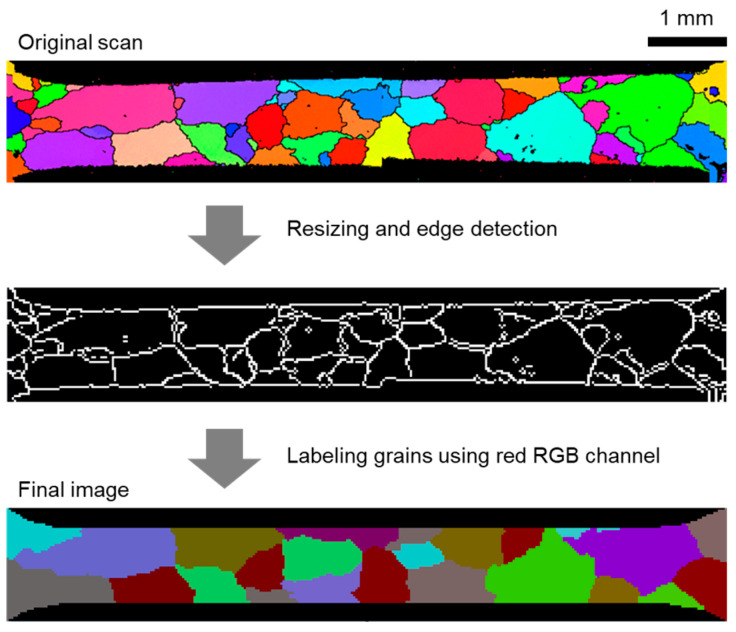
Preparation steps that are applied in order to obtain clear images of top and bottom faces (subsequently used in reconstruction procedure). Different colors indicate different grains.

As was mentioned before, working with a thin oligocrystal specimen that has essentially one layer of grains through the thickness allows us to not only to lower the computational cost but also to obtain the exact arrangement of grains without the need to perform an expensive 3D scan. In this way, the volumetric shape of each grain can be accurately represented, as detailed below, based on its “footprints” at the top and bottom faces of the specimen (Figure 5).

A simple and robust procedure based on a morphing approach that allows such a reconstruction has been developed [39]. The input to the procedure is a set (in our case, a pair) of images that contain matching colors. Each color corresponds to a volumetric phase, e.g., in this case it is a grain. The output is a set of intermediate images which, when stacked on top of each other, form the desired 3D shape. The accuracy of the procedure was successfully verified earlier through destructive testing, which revealed that the predicted grain outlines at various depths from the outer faces were indeed close to the observed ones [39].

As can be seen in Figure 2 and mentioned earlier in Section 2.3, some grains appear only on the top or bottom face, i.e., they do not go all the way through the thickness. Since these grains cannot be omitted and their volumes would leave behind cavities, the reconstruction procedure assumes that these areas are being occupied by the neighboring grains. Furthermore, as can be seen in Figure 5, the EBSD scans of the top and bottom faces of the specimen contain noise. This is seen as small fluctuations of Red–Green–Blue (RGB) values within the same grain, black dots, fuzzy grain boundaries, etc. Since the reconstruction procedure requires colors in the input images to be “clean”, all unwanted noise is removed by the 2 steps illustrated in Figure 5 and explained below.

The first step consists of two substeps. The first substep is to resize the EBSD images (Figure 5, top) to the desired resolution depending on how many finite elements are expected (we picked 316 × 50 for our case). The second substep is to obtain binary (black and white) images containing only the grain boundaries (Figure 5, middle). This was accomplished using the built-in function “EdgeDetect” within the Mathematica computational environment. Since the binary images obtained in this way are still polluted by some residual lines and dots, an additional image cleanup is carried out manually.

The second step is to assign each grain its unique color. A simple fill function of any raster graphics editor (for example, MS Paint) can be used for that purpose. For further mesh generation steps, it is very convenient to keep the information about grain numbers within the image itself. In order to do this, the grain number is stored in a red RGB channel (i.e., green and blue channel values can be chosen randomly). Finally, a script that removes the grain boundaries is applied. The image obtained after these steps is clean, as shown in Figure 5, bottom, and can now be used as an input to the reconstruction procedure described in [39].

**Figure 6:**
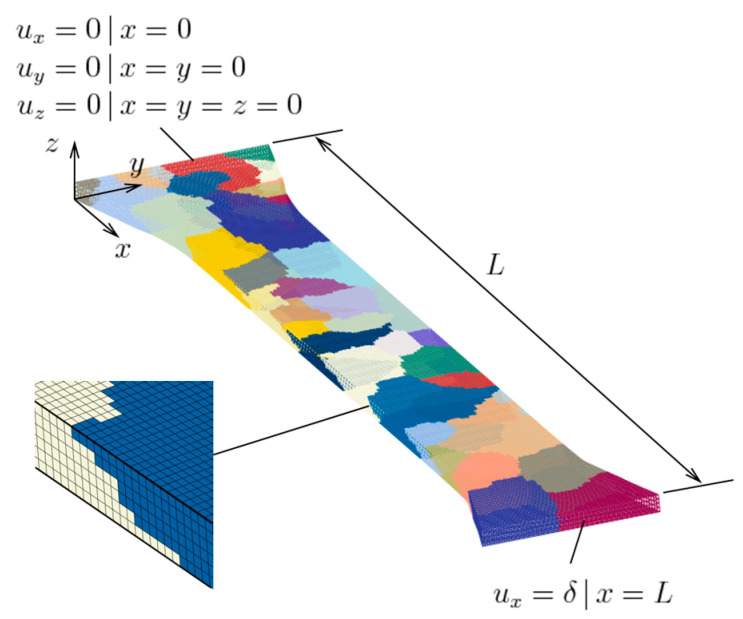
A reconstructed non-columnar voxel model of the oligocrystal specimen and its boundary conditions, also showing the finite element mesh.

The result of applying the reconstruction procedure is a 3D voxel model of the specimen of Figure 2, shown in Figure 6. As can be seen from the perspective view, the 3D shapes of the grains are basically interpolated between their projections at the top and bottom faces. The resolution of the voxel mesh is 316 × 50 × 6, which results in almost 100,000 finite elements (each voxel represents an element). Voxels ensure very good mesh resolution, with the caveat that they do not allow for conformal grain boundaries; instead, these look like a staircase [63]. Due to that, local grain-to-grain fields will be inaccurately represented.

### 3.4. Finite Element Model

This model was created in the commercial, non-linear, implicit code Abaqus/Standard. The element type used is linear with full integration, or C3D8 in Abaqus’ terminology. For the geometric modeling, part of the specimen shoulders, i.e., not only the gauge region, was included. This is to enable the enforcement of boundary conditions; due to deformation-induced roughening, in the plastically deforming regions the flat faces do not remain flat; on the other hand, the material at the shoulders was expected to remain elastic, so this issue was minimized. The boundary conditions implemented are shown in Figure 6, as well. Displacements were prescribed at both ends. In addition, a vertical edge and its adjacent vertex were fixed (i.e., given zero displacements), in order to prevent rigid body motion. As mentioned earlier, the crystal plasticity material model was introduced to the simulation through a UMAT. The only difference from the values mentioned in Section 3.1 and/or listed in Table 2 is the average grain size $d_{g}$ used in Equation (7). While for the identification this was set to 10.3 μm, appropriate for the as-received polycrystal, for the surface roughening simulations it was set to 395 μm, this time appropriate for the oligocrystal in hand.

## 4. Comparison between Experiment and Model

The objective of this work is to assess the predictive capabilities of CPFE by first simulating a specific specimen as realistically as possible and then comparing the numerical results to the experiment performed on that same specimen. In general, common formulations of crystal plasticity models like the one used here, while providing good predictions of the average/polycrystal mechanical response, may be deficient in predicting single or oligocrystal responses [64]. 

### 4.1. Shape of Deformed Specimen

**Figure 7:**
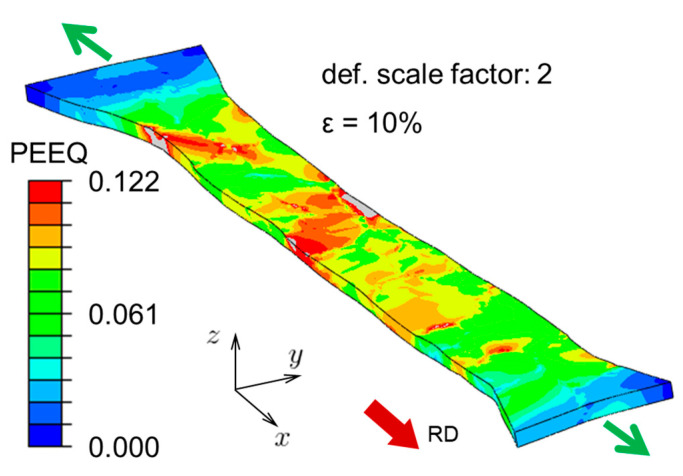
Prediction of the distribution of equivalent plastic strain (PEEQ) in the oligocrystal specimen. The green arrows indicate the direction of tensile loading. A deformation scale factor of 2 is used in order to exaggerate the deformed shape, for easier visualization.

The first feature that can be extracted from the CPFE simulation is the overall shape of the deformed specimen at the final (i.e., 10%) global nominal strain, shown in Figure 7. In that figure, a deformation scale factor of 2 is used to illustrate the post-deformation curvature of the surfaces and edges of the specimen, that initially were flat and straight, respectively. The equivalent plastic strain field, computed from the norm of Equation (4), is also shown in that figure, even though full-field strain measurement techniques (such as digital image correlation) were not used in the experiment, therefore it is not possible to perform a direct comparison. It is, however, interesting to note that the deformation of this oligocrystal specimen is very inhomogeneous, with some areas having experienced significant deformation and others not. This will be revisited in more detail later. It also highlights the difficulty of performing these experiments, as, depending on the grain arrangement and their orientation and proximity to the fillets, in many experiments the deformation was concentrated in a single grain, and no significant roughening could be observed outside of it, rendering them useless for this work. 

**Figure 8:**
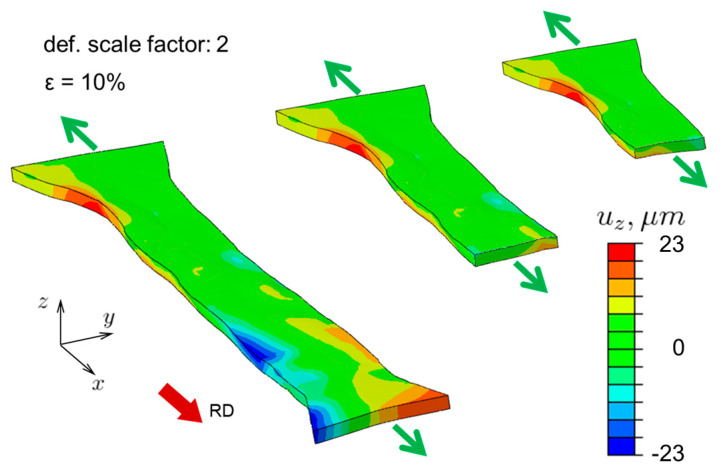
Prediction of the deformed shape of the oligocrystal specimen along with the distribution of z-displacements ($u_{z}$). The green arrows indicate the direction of tensile loading. Two cross-sectional views are shown. A deformation scale factor of 2 is used in order to exaggerate the deformed shape, for easier visualization.

Figure 8 shows the predicted 3D shape and out-of-plane displacement field of the deformed specimen at 10% global nominal strain, with a deformation scale factor of 2 for illustration purposes. We also show two cross-sectional cuts along the gauge area, from which a mild corrugation effect can be observed: the thickness remains more or less constant, while the mid-plane is wavy. While intuition perhaps suggests that the mid-plane should remain flat and thickness should change from grain to grain, it turns out that in our thin specimen, corrugation is more favorable from the equilibrium point of view.

### 4.2. Surface Topography

**Figure 9:**
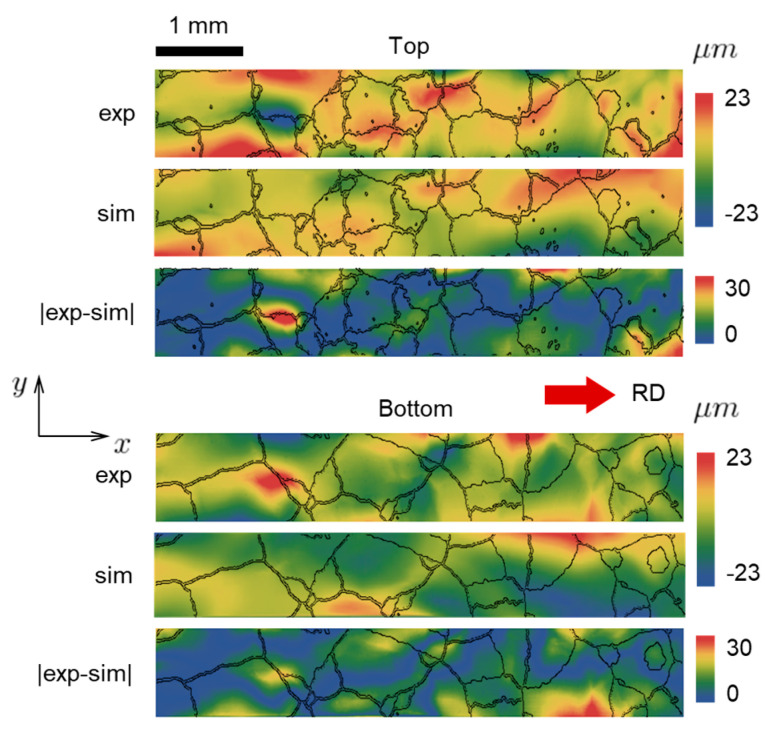
Surface topographies at the top and bottom faces of the specimen, extracted from simulation (marked as “sim”) and experiment (marked as “exp”) at 10% global nominal strain. The absolute difference plots (marked as “|exp-sim|”) are shown, as well.

A quantitative comparison of the surface topography of the deformed specimen from the CPFE model with the experimental results obtained using the laser confocal microscope is given in Figure 9. (Comparison to Figure 1 indicates that Figure 9 shows almost the entire gage section of the specimen.) The comparisons are provided for both the top and bottom surfaces of the specimen. Included in that figure are the outlines of the grains. The absolute difference between model and experiment is also shown in Figure 9. It can be observed that numerous surface features observed experimentally are qualitatively reproduced by the model. In some areas, e.g., those shown in blue in the difference plots, the agreement is quantitative, as well. However, while in this way the absolute difference between simulations and experiments tends to be low, the predicted and measured surface morphologies do not agree perfectly at every location. As this study involves many elaborate steps (both experimentally and numerically), there can be several reasons for such discrepancies. One possible reason is the fact that several grains (mostly small, but a few larger ones as well) appear only in one of the two faces and do not go all the way through the thickness (Figure 2). Since such grains cannot be simply removed from the model (which would result in cavities), the reconstruction procedure assumes that these areas are being occupied by the neighboring grains. That affects the shapes of corresponding grains and therefore it might have also affected the predicted local deformations and hence the resulting surface topography. Another possible reason is that because the specimen is very thin (134 μm) it may have been bent or twisted while being placed into the scanning area for surface elevation measurements. 

One interesting fact that can be observed from Figure 9 is that the peaks and valleys of the surface usually occur in the areas adjacent to grain boundaries and close to the free edges of the specimen. This can be explained as follows. Because the specimen is thin, most of the grains have planar shapes. If we performed a virtual tension test (holding the ends to be coplanar) on an isolated grain (not considering its interaction with neighbors), we would observe that the grain is naturally tilting one way or another due to slip. Once we consider grain interactions, all grain boundaries start acting as kinematic constraints that cause discontinuities in mechanical fields. It can be expected that extreme values of these fields (including displacements and therefore surface elevation) occur at the grain boundaries, not within the grains. This reasoning further explains the mild corrugation effect that can be seen in Figure 8.

**Figure 10:**
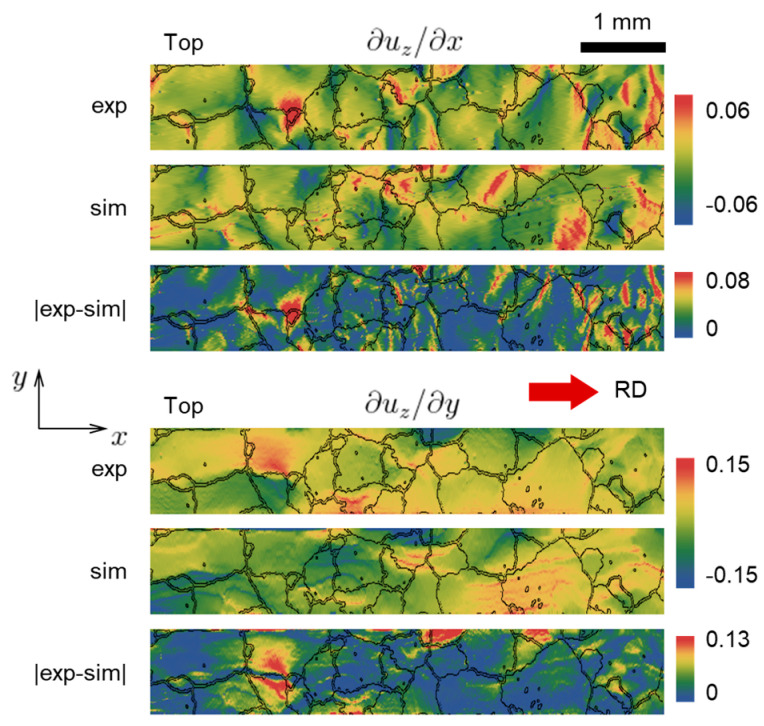
Elevation derivatives at the top face of the specimen, extracted from simulation (marked as “sim”) and experiment (marked as “exp”) at 10% global nominal strain. The absolute difference plots (marked as “|exp-sim|”) are shown, as well.

**Figure 11:**
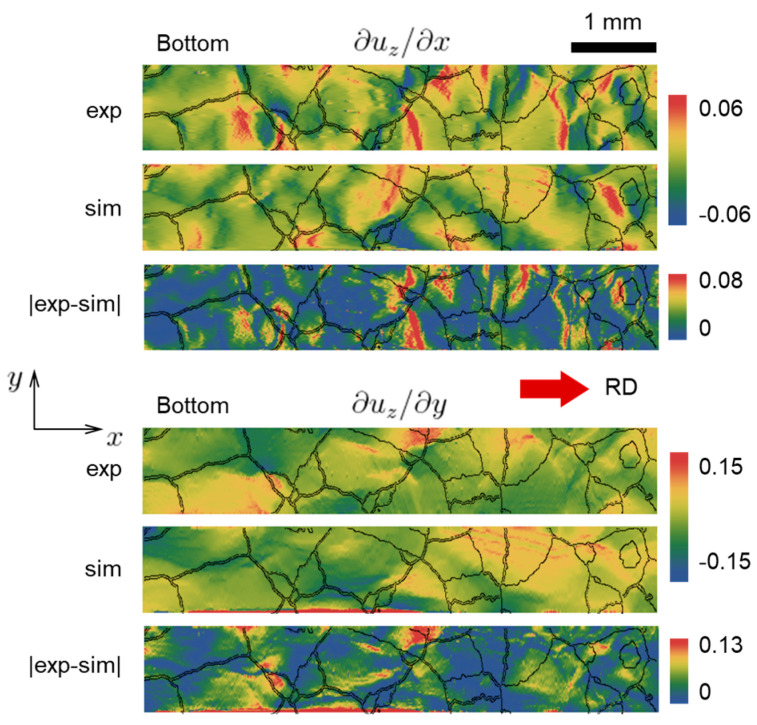
Elevation derivatives at the bottom face of the specimen, extracted from simulation (marked as “sim”) and experiment (marked as “exp”) at 10% global nominal strain. The absolute difference plots (marked as “|exp-sim|”) are shown, as well.

As the direct strain fields from the experiment are not available, in order to estimate the strain-related performance of the CPFE, the surface elevation derivatives$\;\partial u_{z}/\partial x$ and $\partial u_{z}/\partial y$ are shown in Figure 10 and Figure 11, for the top and bottom faces [65]. Note that these derivatives are related to the out-of-plane shear components of the strain tensor. As can be seen from the absolute difference between model and experiment, both derivatives generally match well everywhere except for a few regions.

### 4.3. Average Surface Roughness

**Figure 12:**
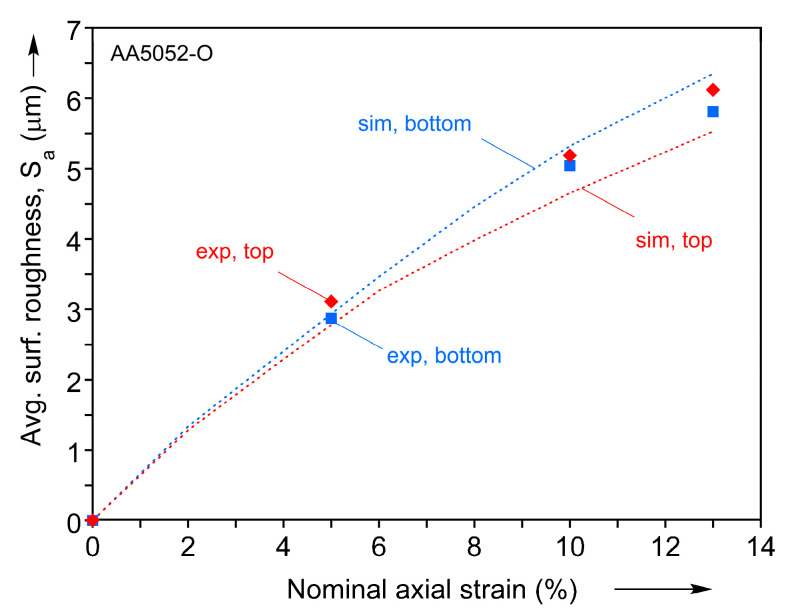
Evolution with the global nominal strain of the average surface roughness $S_{a}$ at the top and bottom faces of the specimen, extracted from simulation and experiment.

The average surface roughness can be defined in the following way:

(13) $$S_{A}=\frac{1}{A}\overset{}{\underset{A}{\int }}\left|z\left(x,y\right)-\bar{z}\right|dA$$

where *A* is the area of interest (i.e., the gage section of the specimen in Figure 1, also reported in Figure 9, Figure 10 and Figure 11) and z(x,y) (see Figure 8) and $\bar{z}$ are the current and average elevation of the surface, respectively. Based on this definition, another result that can be discussed is the evolution of $S_{A}$ with the macroscopically applied axial strain, see Figure 12. Experimental values of $S_{A}$ were calculated by converting images of surface profiles (along with corresponding color bars) into elevation data sets and subsequently applying Equation (13). The dependence obtained is very close to linear, which is in accordance with the previous studies [3,5,7]. As Figure 12 shows, this behavior is quantitatively reproduced very well by the CPFE simulations.

### 4.4. Texture Evolution during Testing

**Figure 13:**
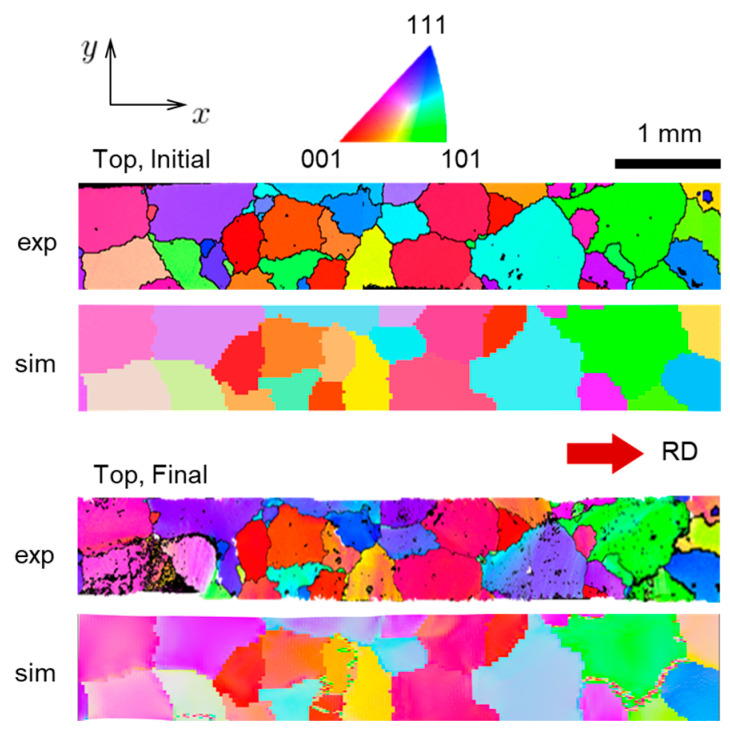
Initial and final (at 10% global nominal strain) grain structure at the top face of the specimen, extracted from simulation (marked as “sim”) and experiment (marked as “exp”).

**Figure 14:**
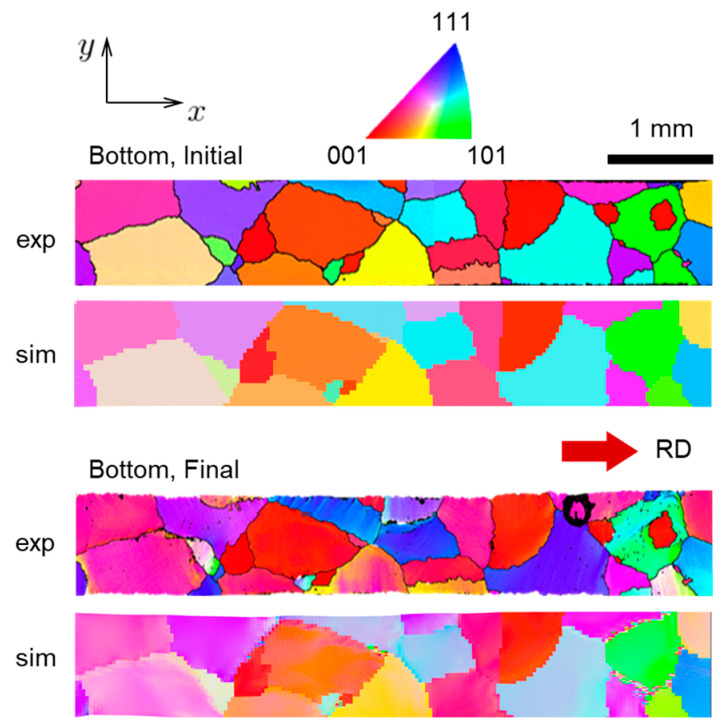
Initial and final (at 10% global nominal strain) grain structure at the bottom face of the specimen, extracted from simulation (marked as “sim”) and experiment (marked as “exp”).

A further assessment of the ability of the CPFE model to represent reality is carried out by comparing the predicted texture to the EBSD measurements of the specimen after the test. During the simulation, the Euler angles are stored at each integration point of the mesh. These Euler angles are visualized with the MTEX toolbox available in MATLAB. As a result, the corresponding Inverse Pole Figure (IPF) color maps before and after deformation and at the top and bottom faces (Figure 13 and Figure 14, respectively) are obtained. Overall, a good agreement between experiment and prediction is observed, with the simulations predicting the texture evolution experienced by the specimen quite well. Of course, since the overall strains are not very high, that evolution is not very strong. Some local disagreements are primarily caused by the fact that the specimen prepared contains several small grains that do not go through the thickness and are only present at one of the faces (as can be seen in Figure 13 and Figure 14). Such grains are not included in the CPFE model and their volume is occupied by neighboring grains instead. In the simulation results, some areas adjacent to grain boundaries contain local regions with random orientations. These random orientations are due to numerical instabilities and are visible as noisy colors (e.g., in the “Top, Final” and “Bottom, Final” images in Figure 13 and Figure 14).

**Figure 15:**
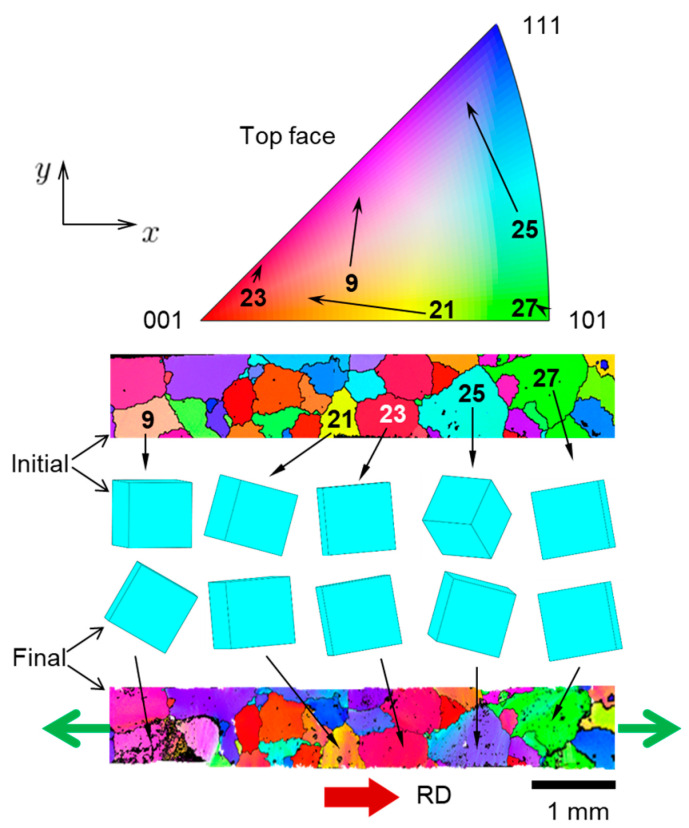
Reorientation of several soft and hard grains (each identified by its number in Table 1) during the experiment, in terms of change in corresponding IPF (RD) colors and of crystal lattice unit cell rotation (presented as cubes).

**Figure 16:**
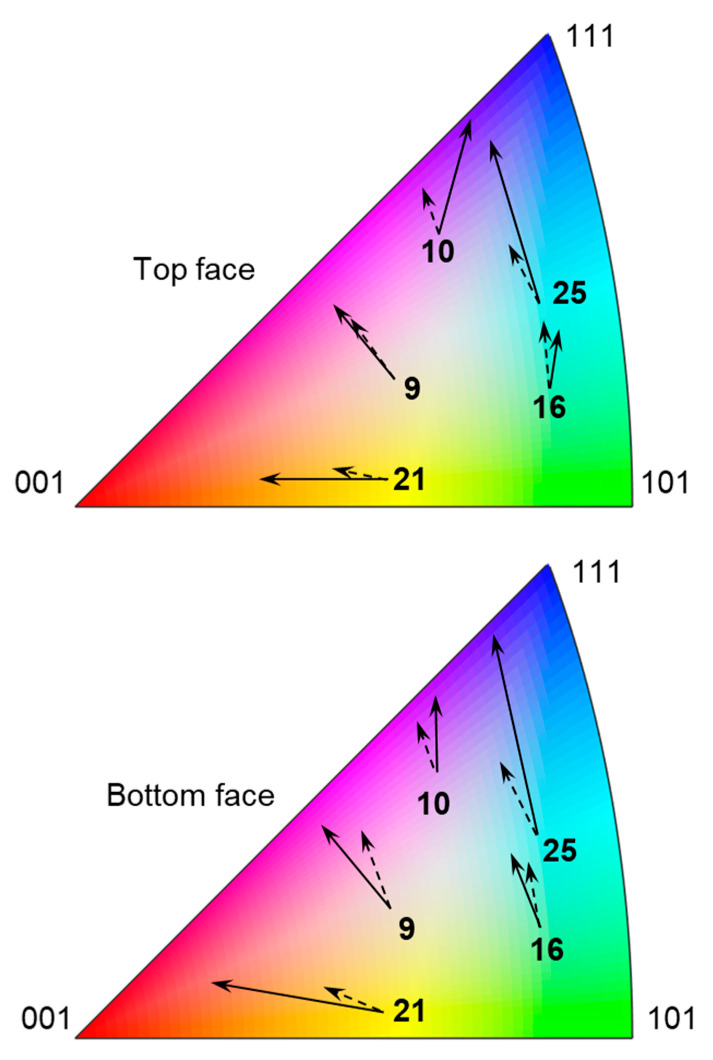
Reorientation of 5 of the softest grains in the oligocrystal specimen (grain numbers identified in Table 1), in terms of change in corresponding IPF (RD) colors. Solid arrows represent experimental observations, dashed arrows represent model predictions.

As a further illustration of the physics of texture evolution, the reorientation (after 10% global nominal strain) of a few “soft” and “hard” grains during deformation is shown in Figure 15. Texture evolution for each grain is represented by a solid arrow in the IPF. Cubes that illustrate the orientation of the unit cell of a given grain, as well as EBSD scans of the entire specimen, are shown with respect to the same global coordinate system. Overall, the applied global strain is not high enough to cause significant grain reorientation in a large scale, but as expected some grains visibly reorient. As can be observed, soft grains (i.e., those with colors that are close to the center of the IPF color map, here grains 9, 21 and 25) are more prone to reorientation [66], while hard grains (i.e., those with colors that are further away from the center, here grains 23 and 27) do not reorient much [66]. In addition, to further examine how the predicted texture matches the measured one, a reorientation graph (after 10% global nominal strain) for the five softest grains in the specimen is shown in Figure 16. In that figure, solid arrows correspond to experimental measurements and dashed arrows to CPFE predictions. An overall agreement between measurements and simulation results is observed visually. To quantify the level of agreement, we introduce the following metric for vectors (arrows) that represents the change in IPF colors:

(14) $$\Delta =\frac{\left|{\vec{a}}_{sim}-{\vec{a}}_{exp}\right|}{\left|{\vec{a}}_{sim}+{\vec{a}}_{exp}\right|}$$

where ${\vec{a}}_{exp}$ and ${\vec{a}}_{sim}$ are vectors representing the measured and predicted IPF color change in Figure 16. When both vectors match perfectly, the value ∆ is zero. When both vectors are of the same magnitude but orthogonal to each other (or when one of the vectors has zero magnitude) the value of ∆ is one. Table 3 provides values of ∆ calculated for each of the 5 pairs of vectors, as well as their average value. In many cases, the values of ∆ are low, indicating that the model captures the experiments well.

**Figure 17:**
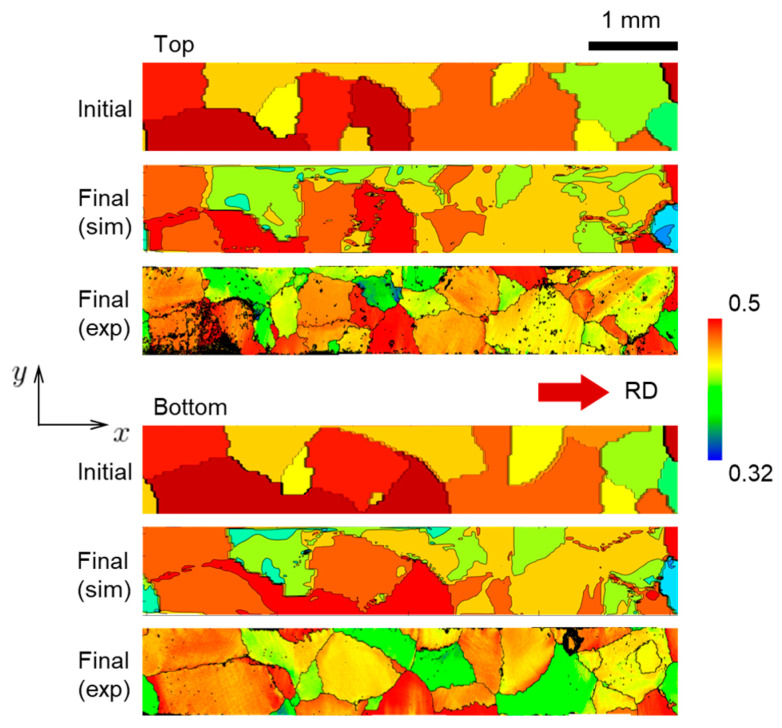
Initial and final (at 10% global nominal strain) distributions of Schmid factor at the top and bottom faces of the specimen, extracted from simulation (marked as “sim”) and experiment (marked as “exp”).

It is also possible to predict the Schmid factor fields (Figure 17). It can be noticed that many distinct grains initially form unions that have very similar values of Schmid factor. These unions separate fully or partially as each individual grain is reorienting in its unique way. Eventually, the grain boundaries within these unions become visible. It can also be seen that initially soft grains that correspond to high initial values of Schmid factor (i.e., closer to red) become harder (i.e., orange/green) as deformation progresses. This fact is in accordance with previous observations of grain reorientations (Figure 15 and Figure 16).

It is interesting to observe that the majority of the grains that have similar initial orientations (similar initial EBSD colors) reorient in the same way. This implies that their reorientation is not affected much by the fact that each of them has quite different arrangements of neighboring grains. One possible explanation for this is that grains in our oligocrystal specimen are more planar than volumetric (since they form a single layer), which means they have more free surface area and therefore they are less restricted by the deformation of neighboring grains.

We can also observe that the CPFE model is able to capture the fact that orientations are varying within the grains (visible as a change in color shades within corresponding grains, e.g., see Figure 13 and Figure 14), which is also seen in EBSD images.

## 5. Analysis of Roughness Statistics

### Modeling Surface Roughening in a Polycrystal

**Figure 18:**
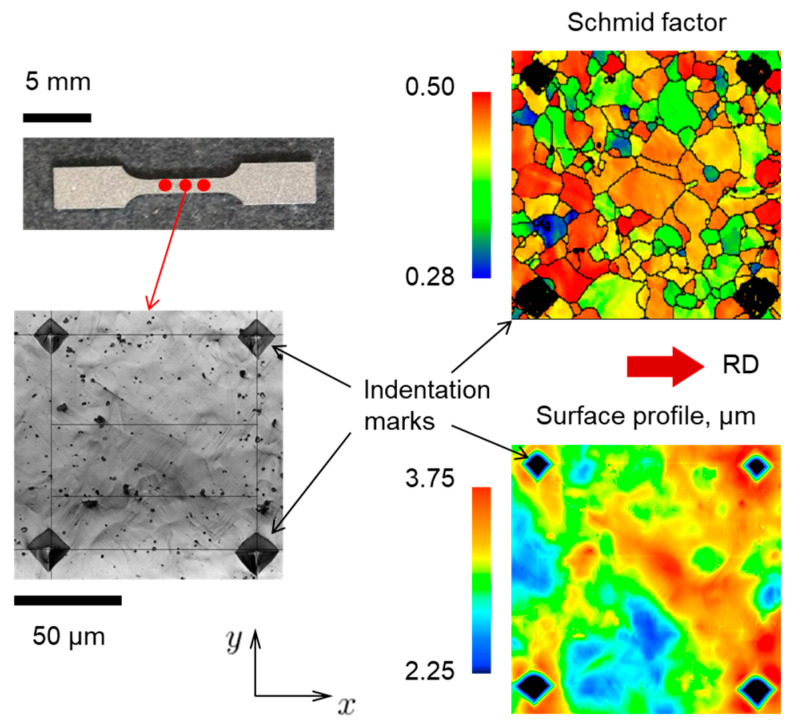
One of the indentation areas from the surface of the polycrystalline specimen, also showing the Schmid factor and surface elevation data extracted from the same area.

**Figure 19:**
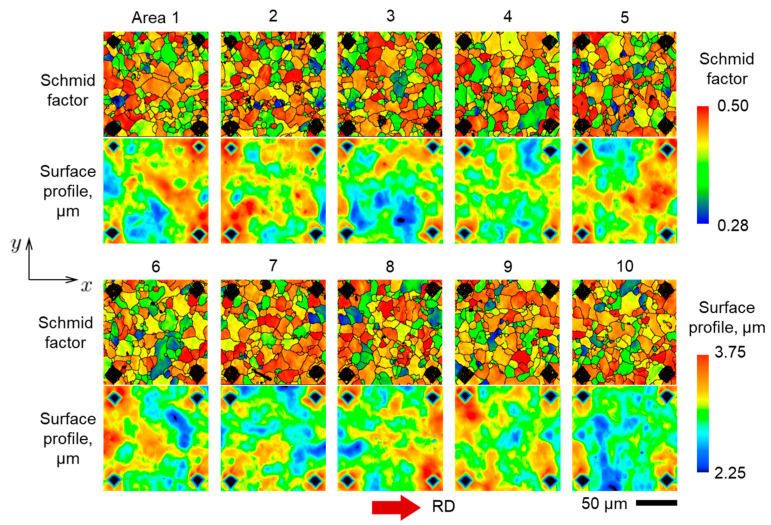
Schmid factor and surface elevation data from 10 (out of 19) marked areas at the surface of the polycrystalline specimen. The color legends are the same as for Figure 18.

**Figure A1:**
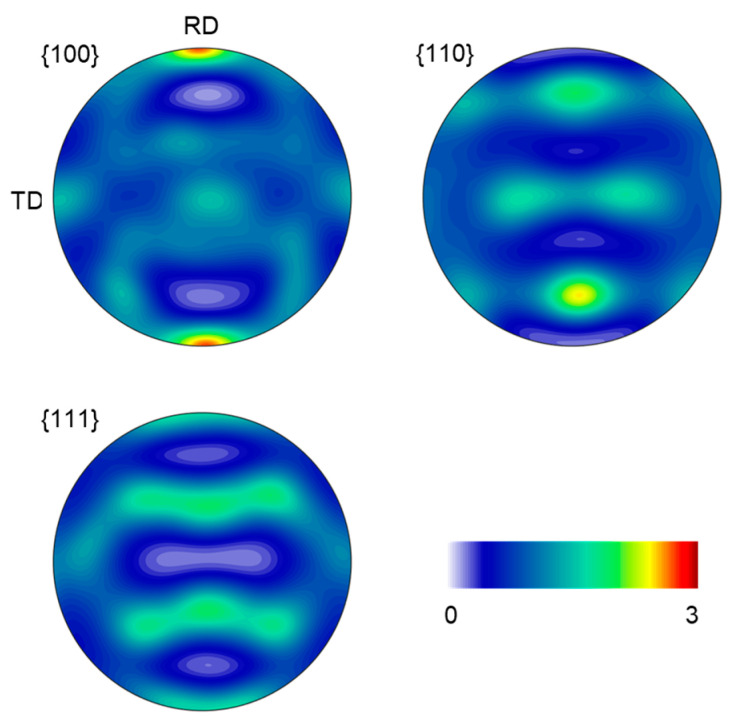
Crystallographic texture of as-received polycrystalline AA5052-O, shown as a set of pole figures.

In order to further verify the CPFE model, we analyze its performance for the case of polycrystal AA5052-O. In addition, the study of the polycrystal reveals statistical patterns that are hard or impossible to conclude from oligocrystal results. A tensile specimen of the same geometry as Figure 1 is analyzed, but with no heat treatment applied, i.e., in the as-received, polycrystalline state (the pole figures of corresponding texture are shown in Figure A1). To assist with data extraction, 19 sets of indentation marks were applied to one of the faces of the specimen (Figure 18). These marks provide a useful reference as they appear both in texture and surface elevation scans (after a 10% global nominal prestrain), see Figure 18. Data from 10 of the 19 areas marked by the indentations are shown in Figure 19.

**Figure 20:**
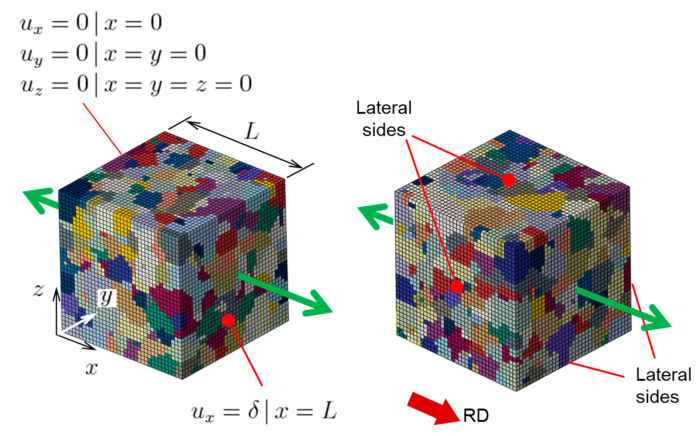
Finite element meshes and boundary conditions (L is the side of the cube) for two Representative Volume Elements (RVEs) that were used to model the roughening behavior of a polycrystal. Different colors represent different grains.

In order to model the roughening behavior of this polycrystalline specimen, two Representative Volume Elements (RVEs) are constructed using the DREAM.3D package, a digital representation environment for the analysis of 3D microstructures [67]. The average grain size of equiaxed grains ($d_{g}$ = 10.3 μm) is provided as the only input to DREAM.3D. The RVE models are created using Abaqus/Standard, as in the rest of this work. Each RVE contains about 1300 grains, discretized into 35 × 35 × 35 ≈ 43,000 finite elements. The element type is linear with full integration (C3D8). The meshes adopted are deemed statistically sufficient, as will be verified below by comparing the results of the two RVE models. Each RVE has the same initial texture as the polycrystal (Figure A1). Boundary conditions for both RVEs are the same and are shown in Figure 20. It should be noted that these boundary conditions provide shear-free surfaces, which is somewhat less restrictive than being embedded in a polycrystal. The possible effect of this will be discussed below. The crystal plasticity model described above is used in the simulations, using a UMAT. 

**Figure 21:**
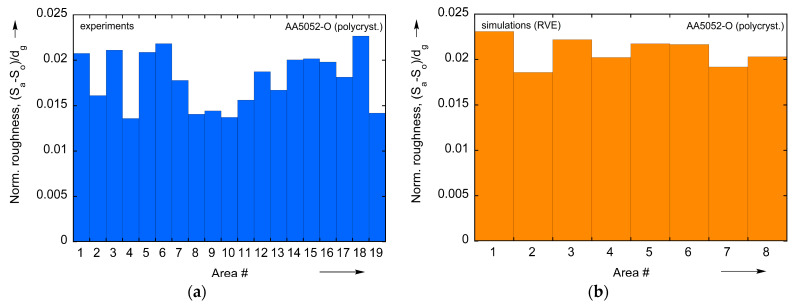

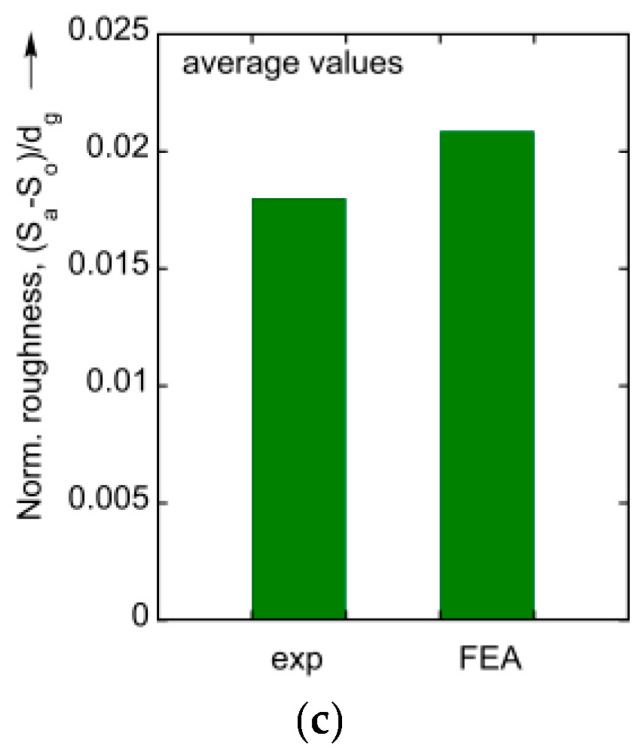
A comparison between experiment and model for a polycrystal: raw values of normalized roughness parameters extracted from corresponding regions in (**a**) experiment (19 areas) and (**b**) model (8 areas, 4 from each RVE). (**c**) Averages of experiment and mode2. Texture–Surface Morphology Relations.

By loading the two RVEs in tension, the roughness metric $S_{a}$ is extracted from the 8 lateral sides (4 from each one). At the same time, $S_{a}$ is calculated experimentally, for each of the 19 areas of the tensile specimen. In order to make a fair comparison between CPFE results and experiment, the initial roughness value of the specimen, $S_{0}$, is subtracted. Also, the result is divided by the average grain size $d_{g}$, since the roughness is known to be linearly proportional to $d_{g}$. The value of $d_{g}$ = 10.3 μm is calculated using the ASTM standard E112-13 [47], see Section 2.2. Normalized values of surface roughness $\left(S_{a}-S_{0}\right)/d_{g}$ from experimental data (19 marked areas) and two RVEs (8 lateral sides) at 10% global nominal strain are shown in Figure 21a, b, respectively. As could be expected, there is quite a bit of variation between different regions since each one contains a relatively limited number of grains. The CPFE results show somewhat greater roughening than the experiments, perhaps because of the shear-free boundaries adopted in the model. Nevertheless, the average values of experiment and simulation are not that far off, which is seen in Figure 21c. This study substantiates the conclusions drawn from the oligocrystal, i.e., that the present constitutive framework is capable of capturing the roughening phenomena in an average sense, despite non-perfect agreement with the experiments in some locations.

Returning now to the experiments, since we have access to a considerable amount of data that can be extracted from the 19 areas of the polycrystalline specimen, we attempt here to find some mathematical relation between texture and the resulting surface morphology. It should be noted that the motivation here is not a further validation of the CPFE modeling framework; rather, this effort is intended to provide a new look at experimental observations and to contribute to production of sheets with reduced propensity for roughening. 

It is important that indentation marks appear both on EBSD scans and surface topography maps (Figure 19), which ensures a correspondence between texture and surface elevation before and after deformation. From each individual grain, we extract several non-dimensional data sets:

1. Ratio of average elevation within the grain ($\delta =\frac{1}{A_{i}}\int_{A_{i}}^{}zdA$, where $A_{i}$ is the area of the given grain) to the average surface roughness of the specimen ($S_{A}$, see Equation (13)):
  (15) $$\eta =\frac{\delta }{S_{A}}$$
2. Ratio of average roughness within the grain ($S_{i}=\frac{1}{A_{i}}\int_{A_{i}}^{}\left|z-\bar{z}\right|dA$) to the average surface roughness of the specimen ($S_{A}$):
  (16) $$ℜ=\frac{S_{i}}{S_{A}}$$
3. Average Schmid factor of the grain (SF) with respect to the loading axis.
4. Relative grain size:
  (17) $$\gamma =\frac{r}{\bar{r}}$$
  where r is an effective radius of the given grain $r=\sqrt{A_{i}/\pi }$ and $\bar{r}$ is the average effective radius among all grains.

**Figure 22:**
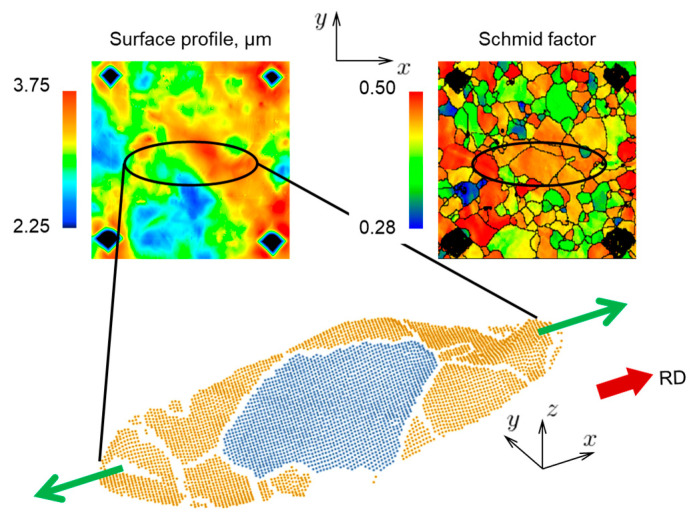
Surface topography and Schmid factor data, extracted from a grain and its elliptical neighborhood. Similar data are extracted from each surface grain of the polycrystalline specimen (e.g., Figure 19 and Figure 20).

It is also important to consider the effect of the surrounding grains. Assuming that the surrounding grains that are located along the loading axis have the greatest effect on the deformation of a given grain, we construct around each grain a circular or elliptical neighborhood, from which we extract $\eta_{n}$, ${ℜ}_{n}$ and $SF_{n}$ correspondingly. Note that the quantities with the subscript “n” are for neighbors, while the quantities with no subscript are for a given grain. As an example, one chosen grain and its neighborhood (here, n = 11) are shown in Figure 22. As a result, from each individual grain (about 1300 grains in each RVE) we obtain 7 parameters (ℜ, ${ℜ}_{n}$, η, $\eta_{n}$, SF, $SF_{n}$, γ) and establish their ranges and distributions. Our assumption is that these parameters are sufficient for correlating the surface roughness to (surface) texture features. Our goal then is to find some relation between these parameters in the form:

(18) $$\eta =f\left(ℜ,{ℜ}_{n},\eta_{n},SF,SF_{n},\gamma \right)$$

where f is an unknown function. In order to find f, we perform a symbolic Monte Carlo search by generating a very large number of random symbolic expressions and then pick the ones that show the best fit. We perform the search with the following parameters: unary operations $x^{2}$, $\sqrt{\left|x\right|}$, ln|x|, exp(x); binary operations +, −, ×, ÷; and constants 2 and 3. That specific set of operations includes some of the most commonly used in mathematical relations (minus trigonometry ones, which do not seem to be relevant here). Hence, we feel that they should be sufficient to express the unknown formula that we are seeking. The maximum number of math operations per expression is set to 10, to limit the complexity of these formulas. That number was found by experimentation, and by visually inspecting the resulting formulas. The total number of unique generated random relations is around 7 million, which was also found to be sufficient through experimentation.

**Figure 23:**
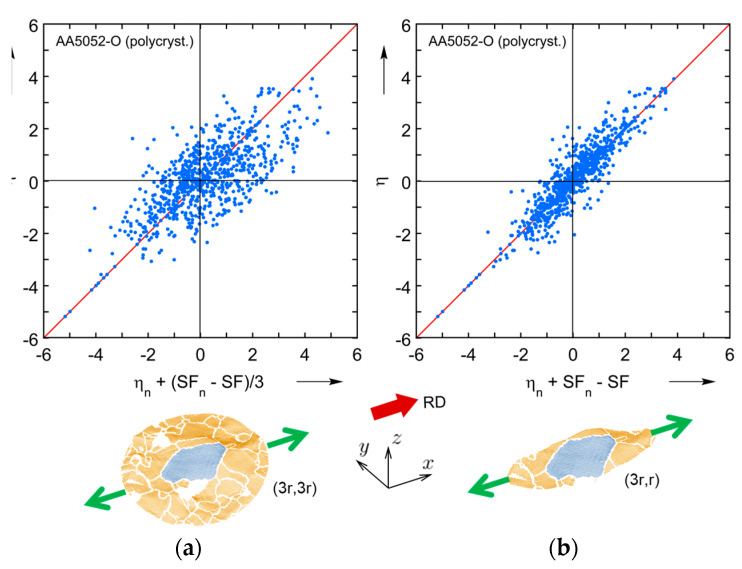
Correlations shown for (**a**) circular (radius 3r) and (**b**) elliptical (semi-axes 3r and r) neighborhoods. Red solid line corresponds to perfect correlation; blue dots are actual observations. The elliptical neighborhood offers better correlation (variance = 0.16) than the circular one (variance = 0.49).

Several different shapes of neighborhood are investigated and are shown along with the corresponding identified relations in Table 4. The strongest correlation is obtained for neighborhoods that are elongated and aligned with the loading axis; specifically, for an elliptical neighborhood with semi-axes 3r and r (as well as 2r and r). A comparison between correlations obtained using elliptical and circular shapes of the neighborhood is shown in Figure 23. The elliptical neighborhood is seen to offer a better correlation than the circular one. The texture–morphology relation obtained for elongated neighborhoods has the following form:

(19) $$\left(\eta -\eta_{n}\right)\propto (SF_{n}-SF)$$

or, in a form similar to Equation (18):

(20) $$\eta =\eta_{n}+SF_{n}-SF$$

Given the great number of iterations (7 million), Equation (20) has statistical significance. Indeed, the code was run multiple times and this equation was consistently found to be the best.

Since the Schmid factor characterizes the softness or hardness of a given grain, the physical meaning of this relation can be understood as follows: the difference between the elevation of a given grain and its neighborhood is proportional to the difference between their corresponding Schmid factors. In other words, if a grain and its surroundings are equally soft or equally hard, a large change in elevation should not be expected. In contrast, a hard grain embedded into a soft neighborhood (as well as a soft grain embedded into a hard neighborhood) will cause a more noticeable change in elevation. Also, as can be seen from the scatter in Figure 23, the obtained relations are not applicable to individual grains, but instead capture the overall trend. Since the effect of subsurface grains is not taken into account in this analysis, that could be responsible for some of the scatter shown in Figure 23. Furthermore, not taken into account in this analysis is the possible existence of clusters of grains with similar crystallographic orientations, which can give rise to phenomena such as ridging and roping [22].

In comparison to current understanding of surface roughening, this section statistically identifies a specific mathematical relation between non-dimensionalized texture and morphology parameters, after checking a very large amount of candidate relations. It can be said that, at least from a surface roughening point of view and considering only the effects of the surface grains (e.g., not grain size, subsurface grains, etc.), it is advantageous to have grains of similar orientations, rather than a randomly textured polycrystal. A similar analysis has been performed for the effect of properties of neighboring grains on intra-granular misorientation development in a given grain with plastic strain [68]. It was found that smaller intra-granular misorientation levels are associated with “softer” neighborhoods and vice vers

## 6. Summary and Conclusions

In this work, we studied the deformation-induced surface roughening of an Al–Mg oligocrystal (produced from polycrystalline AA5052-O) by examining the roughening behavior of a mesoscale-size specimen that contains relatively few grains, most of them arranged in a single layer. The specimen was deformed plastically under (macroscopically) uniaxial tension. The initial and final textures of the specimen, as well as surface topographies at the top and bottom faces, were measured. The physics of plastic deformation were captured with an appropriate CPFE model. Four unknown material parameters were determined inversely using an efficient black-box optimization procedure. Differently from other numerical studies on deformation-induced surface roughening, the FE model created in this work had realistic (non-columnar) shapes of the grains. These shapes were reconstructed from EBSD scans of top and bottom faces using a custom-developed shape interpolation procedure that is based on a morphing approach. Using this FE model, we were able to analyze a number of aspects such as the deformed shape of the specimen, surface topographies at top and bottom faces, corresponding elevation derivatives, evolution of the average roughness value with straining, texture and Schmid factor after deformation and reorientation of several soft and hard grains. The results extracted from the model match fairly well with experimental observations, certainly in an average sense, and with only local deviations.

In order to further verify the CPFE model, we also analyzed statistical aspects of surface roughening by simulating the behavior of a polycrystal. A tension test was performed on a polycrystalline AA5052-O specimen. Two representative volume elements (each containing around 1500 grains) with texture corresponding to the initial one were constructed and their deformation was simulated. A good match between average roughness values from the model and from the experiment was observed. The polycrystal specimen contained a number of indentation marks on its surface, so that it was possible to establish the correspondence between grain texture and morphology at these marked regions. Subsequently, using an automated method based on a symbolic Monte Carlo approach we were able to identify relations between the problem variables. 

After analyzing the behavior of oligocrystal, several conclusions can be drawn. The CPFE model is overall capable of capturing the deformation-induced roughening behavior of this material. This is certainly true in an average sense, but also to some extent in a local sense, with the exception of only a few regions of the specimen. Some limited corrugation of the specimen—bending of the mid-plane with the thickness remaining relatively constant—was observed and was also captured by our model. In addition, peaks and valleys of surface topography were observed primarily at the grain boundaries, which is due to the fact that grain boundaries act as kinematic constraints and therefore extreme values of mechanical fields such as out-of-plane displacement also appear there. Finally, grain reorientations seem to depend mostly on the initial orientations of these grains, and not on arrangement/orientations of their neighbors, owing to the pancake-like shapes of grains in our oligocrystal which are not very restrictive kinematically.

From the polycrystal analysis, we can conclude that a model with proper statistical input such as initial texture and average grain size is enough to capture the roughening behavior with good accuracy. In addition, it was shown that simple relations between the Schmid factor and the non-dimensional surface elevation for individual grains and their surface neighbors can be inferred purely from experimental data and also can be easily explained from the physical point of view. On the other hand, the analysis presented here does not take into account phenomena such as clustering of grains with similar orientations, which has been shown to affect the roughening behavior of some materials, including phenomena such as ridging and roping (e.g., [22]). Future work will advance the CPFE model into a strain gradient plasticity (SG-CPFE) formulation [69] to evaluate whether the SG-CPFE model will improve the local predictions of the mesoscopic mechanical response and resulting roughness fields.

## Figures and Tables

**Table 1:** Initial grain orientations in the oligocrystal. Bunge Euler angles ($\varphi_{1}$, Φ, $\varphi_{2}$) in degrees are listed.

Grain Number	$\varphi_{1}$	Φ	$\varphi_{2}$
1	343.1	36.2	58.7
2	170.4	7.5	78.5
3	155	30.6	3.6
4	122.1	37.9	0.5
5	37.9	23.7	84.3
6	342.9	20.6	81.4
7	33.1	23.1	56.9
8	44.3	43.6	37.9
9	192	38	80.8
10	235.8	38.7	38.9
11	227.7	32.4	36.1
12	322.2	30.7	46.8
13	82.8	25.7	30.8
14	247.4	26.5	14.3
15	328.5	41.5	54.4
16	215.5	11.4	23.8
17	354.9	42.7	5.6
18	169.4	19.3	83.3
19	258.1	51	45.5
20	340.4	36.1	17.9
21	233.7	48.4	58.1
22	209.9	41.4	7.1
23	38.5	25.7	15
24	185.8	16.9	60.4
25	37.7	14.8	40.8
26	202.9	29.6	76.9
27	7.3	33.8	83.9
28	89.1	14.4	53.3
29	302.1	19.6	76.5
30	352	10.7	54
31	283.2	24.4	9
32	314.7	33.3	36.4
33	229.9	28.8	84.7
34	181	43.6	53.4
35	77.8	51	53.2
36	337.2	15.8	48.8
37	154.4	10.2	7.2
38	263.7	6.1	6.9

**Table 3:** Level of agreement between predicted and measured reorientations.

Grain Number	Δ (Top)	Δ (Bottom)
9	0.12	0.20
10	0.49	0.26
16	0.13	0.11
21	0.36	0.43
25	0.42	0.42
Average	0.29	

**Table 4:** Relations constructed for different shapes of the neighborhood.

Shape	Semi-Axes	Relation
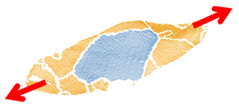	(3r,r)	$\eta =\eta_{n}+SF_{n}-SF$
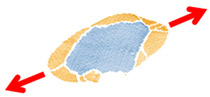	(2r,r)	$\eta =\eta_{n}+SF_{n}-SF$
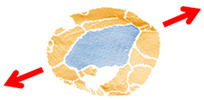	(2r,2r)	$\eta =\eta_{n}+\frac{SF_{n}-SF}{2}$
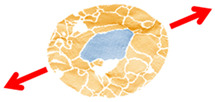	(3r,3r)	$\eta =\eta_{n}+\frac{SF_{n}-SF}{3}$

## Data Availability

The data presented in this study are available on request from the corresponding author.

## Appendix A

The texture of the as-received polycrystalline AA5052-O is shown in Figure A1. In the pole figures presented, we can observe patterns typical of a rolled texture, such as two parallel features in the {111} pole figure. In addition, we can see the presence of cube texture (pronounced poles at top, bottom, left, right) in the {100} pole figure, which is due to recrystallization.
